# Dendritic Cell-Based Immunotherapy of Acute Myeloid Leukemia

**DOI:** 10.3390/jcm8050579

**Published:** 2019-04-27

**Authors:** Heleen H. Van Acker, Maarten Versteven, Felix S. Lichtenegger, Gils Roex, Diana Campillo-Davo, Eva Lion, Marion Subklewe, Viggo F. Van Tendeloo, Zwi N. Berneman, Sébastien Anguille

**Affiliations:** 1 Laboratory of Experimental Hematology, Vaccine & Infectious Disease Institute, Faculty of Medicine & Health Sciences, University of Antwerp, 2610 Wilrijk, Antwerp, Belgium; heleen.vanacker@uantwerpen.be (H.H.V.A.); maarten.versteven@uantwerpen.be (M.V.); gils.roex@uantwerpen.be (G.R.); Diana.CampilloDavo@uantwerpen.be (D.C.-D.); eva.lion@uza.be (E.L.); vigske@gmail.com (V.F.V.T.); zwi.berneman@uza.be (Z.N.B.); 2 Department of Medicine III, LMU Munich, University Hospital, 80799 Munich, Germany; F.Lichtenegger@gmx.de (F.S.L.); Marion.Subklewe@med.uni-muenchen.de (M.S.); 3 Division of Hematology and Center for Cell Therapy & Regenerative Medicine, Antwerp University Hospital, 2650 Edegem, Antwerp, Belgium

**Keywords:** dendritic cells, immunotherapy, acute myeloid leukemia

## Abstract

Acute myeloid leukemia (AML) is a type of blood cancer characterized by the uncontrolled clonal proliferation of myeloid hematopoietic progenitor cells in the bone marrow. The outcome of AML is poor, with five-year overall survival rates of less than 10% for the predominant group of patients older than 65 years. One of the main reasons for this poor outcome is that the majority of AML patients will relapse, even after they have attained complete remission by chemotherapy. Chemotherapy, supplemented with allogeneic hematopoietic stem cell transplantation in patients at high risk of relapse, is still the cornerstone of current AML treatment. Both therapies are, however, associated with significant morbidity and mortality. These observations illustrate the need for more effective and less toxic treatment options, especially in elderly AML and have fostered the development of novel immune-based strategies to treat AML. One of these strategies involves the use of a special type of immune cells, the dendritic cells (DCs). As central orchestrators of the immune system, DCs are key to the induction of anti-leukemia immunity. In this review, we provide an update of the clinical experience that has been obtained so far with this form of immunotherapy in patients with AML.

## 1. Introduction

Acute myeloid leukemia (AML) is a highly aggressive type of leukemia characterized by the uncontrolled clonal proliferation of abnormal myeloid cells in the bone marrow [1,2,3]. AML primarily affects older people; over 50% of AML patients are older than 65 [1,2]. The past decades have witnessed an increased incidence of AML, which is mainly due to the aging population [4]. Treatment of AML remains challenging, although considerable advances have been made over the last 50 years. In this context, an influential discovery in the treatment of AML was the cytarabine-based chemotherapy in association with an anthracycline or related agent during the 1970s. This combination chemotherapy regimen significantly improved the probability to induce complete remission (CR) [5] and has consequently remained the backbone of frontline AML therapy [6]. The next major development in the treatment of AML was marked by the implementation of allogeneic hematopoietic stem cell transplantation (HSCT), which transformed this disease into a potentially curable one [7].

Despite these accomplishments, the long-term outcome of adults with AML remains precarious, with a five-year overall survival (OS) rate hovering at around 25% [8]. According to data from the Surveillance, Epidemiology, and End Results (SEER) Program of the National Cancer Institute (NCI, Bethesda, MD, USA), the steady improvement in long-term survival since the mid-1970s is almost completely attributable to the decrease in mortality among patients younger than 65 years. By contrast, the prognosis of patients aged 65 years or older has not improved considerably over time, the latest reported five-year survival rate being <10% [8]. Considering the above observation that elderly patients represent a significant and rising proportion of AML patients [4], one cannot but conclude that the overall picture remains grim, and that the scientific and therapeutic progress made did not translate into an equivalent improvement in long-term survival [2].

Perhaps the most important reason for this unsatisfying outcome is the high relapse-rate in leukemia, especially in elderly AML patients. Indeed, up to 80% of the patients older than 60 years will eventually relapse, despite having initially achieved CR with conventional (poly)chemotherapy [9,10]. It is generally accepted that this relapse arises from the existence of a small reservoir of treatment-resistant leukemic (stem) cells (LSCs) that persist after chemotherapy [11], a condition known as minimal residual disease (MRD), which may evolve to a full clinical relapse [12,13]. Allogeneic HSCT can be used effectively to clear MRD and has a positive impact on relapse rate and survival. Unfortunately, HSCT is still associated with significant morbidity and mortality, generally limiting its use to younger patients with fewer co-morbidities [2]. For patients with no transplant donor available or for older patients who are usually deemed unfit for HSCT, there is currently no standard post-remission therapy to control MRD and avoid relapse [1,4,6,14].

**Figure 1:**
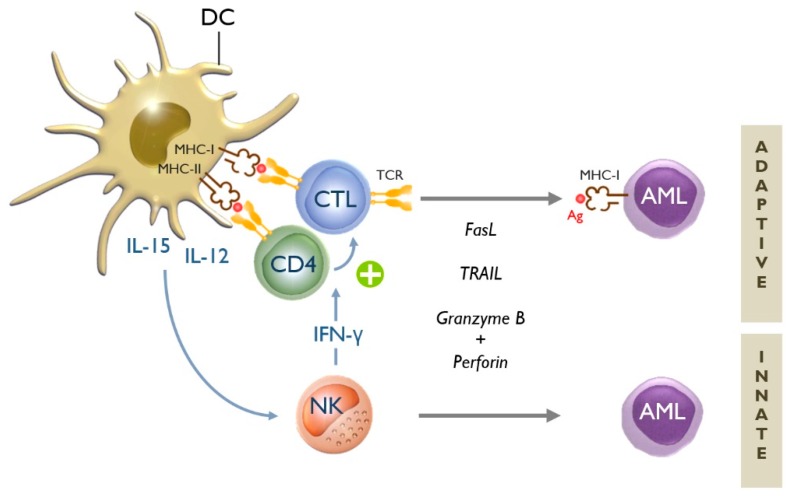
Dendritic cells are key to the induction of adaptive and innate anti-leukemia immunity. Dendritic cells (DCs) can stimulate both adaptive and innate immune responses against acute myeloid leukemia (AML) cells [12]. There exist two types of adaptive immune responses: humoral or B-cell-mediated (not shown in this figure), and cellular or T-cell-mediated immune responses. The initiation of the latter type of immune response involves the presentation of AML-related antigens (Ag) by DCs via major histocompatibility complex (MHC) class I and II molecules to CD8^+^ cytotoxic T-lymphocytes (CTLs) and CD4^+^ helper T cells, respectively. If appropriately stimulated, naive CD4^+^ T cells (T_H_0) can be polarized into T helper type 1 (T_H_1) cells, which in turn support the generation of antigen-specific CTLs (+). These CTLs—via their specific T-cell receptor (TCR)—are capable of recognizing AML cells that display the particular antigen(s) to which the CTLs are directed [12]. CTLs possess different tools in their armamentarium to kill AML cells, such as death receptor ligands (e.g., Fas ligand (FasL) and tumor necrosis factor-related apoptosis-inducing ligand (TRAIL)) and cytolytic proteins released from intracytoplasmic granules (e.g., granzyme B and perforin) [16]. The innate arm of the immune system is unequivocally important for mounting effective anti-leukemia immunity [20]. Innate effector cells, predominantly natural killer (NK) cells, are able to eradicate AML cells in a non-antigen-specific, non-MHC-restricted manner. NK cells can utilize the same cytolytic tools as CTLs [16]. In addition to their direct cytotoxic activity, NK cells also play an immunoregulatory role by secreting cytokines such as interferon (IFN)-γ. Through this so-called “helper” function, NK cells can support the generation of T_H_1 and CTL responses [21,22]. Several DC-derived cytokines are known to be involved in DC-mediated NK-cell activation, including interleukin (IL)-12 and IL-15 [23]. The latter is usually not secreted by the DCs, but “trans-presented” on the DC surface via IL-15 receptor-α [24,25].

The above observations emphasize the need for more effective and less aggressive treatment alternatives to improve the long-term outcome of AML, especially in elderly patients. It is within this context that immunotherapy has come to the fore in recent years [12,15,16,17]. From the experience with allogeneic HSCT, we have learned that immune cells are indeed capable of recognizing and eliminating AML cells—the so-called “graft-versus-leukemia” (GvL) effect [12,16]. Leukemia antigen-specific CD8^+^ cytotoxic T-lymphocytes (CTLs) and natural killer (NK) cells are the main immune effector cells responsible for attacking and killing AML cells (Figure 1) [12]. As conductors of the immunological orchestra, dendritic cells (DCs) are endowed with the potent and unique ability to harness the anti-leukemia activity of both immune effector cell types. It is therefore not surprising that DCs have attracted much interest in recent years as tools for immunotherapy of AML [12,18,19]. In this review, we summarize the clinical experience that has been obtained with this form of immunotherapy in AML.

## 2. Clinical Use of DCs for Immunotherapy of AML

The feasibility, safety/toxicity and immunogenicity of DC vaccination in AML has been reviewed elsewhere (see Ref. [26]) and are outside the scope of this review. In addition, our group has previously done a cost–benefit analysis and found that DC therapy following chemotherapy is a cost-effective treatment [27]. Table 1, Table 2 and Table 3 provide an overview of all DC-based clinical studies performed so far in AML. As of 31 December 2018, nearly 200 patients with AML have been treated with this form of immunotherapy.

As shown in Table 1, Table 2 and Table 3 most studies have relied on DCs derived from autologous peripheral blood monocytes (moDCs), although allogeneic DCs have also been used [26]. In some studies, autologous leukemic blast cells were used as precursor cells for DC generation (AML-DCs) [26]. In one clinical trial, AML-DCs were generated from a leukemic cell line [28]. One drawback of the use of AML-DCs is their limited yield compared to moDCs, making clinical implementations more cumbersome [29]. In addition, in a head-to-head comparison between AML-DCs and moDCs, Draube and colleagues [29] found that moDCs were more effective in activating autologous leukemia-specific T cells than AML-DCs. Several arguments exist that could explain these findings. It has been postulated that AML-DCs lack the expression of 4-1BBL, an important ligand for co-stimulation [30]. Alternatively, indoleamine 2,3-dioxygenase 1 (IDO-1) expression by leukemic blasts can result in DCs with a more tolerogenic functionality [31]. Combined, these findings provide a preference of the use of moDCs over AML-DCs. On the other hand, AML-DCs have the advantage over moDCs in the sense that they present the full antigen repertoire of the leukemic blasts from which they are derived, thereby obviating the need for an antigen-loading step [26]. MoDCs, by contrast, require to be loaded with one or more AML antigens. This can be done by exogenous pulsing with a peptide (e.g., Wilms’ tumor 1 [WT1] peptide) [32,33,34,35,36], by pulsing with apoptotic AML cells or lysates [37,38,39,40], by fusing the DCs with leukemic blasts (so-called fusion hybrids) [41,42], or by messenger RNA (mRNA) electroporation [8,43,44,45]. Messenger RNA electroporation involves the application of a brief electrical pulse to make the DC plasma membrane transiently permeable allowing the antigen-encoding mRNA to enter the cytosol. The mRNA will then be translated by the DCs into full-length antigenic protein. The translated antigen is further degraded into small peptide fragments, which are presented on the DC surface via major histocompatibility complex (MHC) molecules to the T cells. This technique has been used to load moDCs with one of the following leukemia-associated antigens: WT1, human telomerase reverse transcriptase (hTERT) and preferentially expressed antigen in melanoma (PRAME) [8,43,44,45]. mRNA electroporation is a non-viral gene transfer method; only one study implemented an (adeno)viral transduction approach for gene transfer of the leukemia-associated antigens survivin and MUC1 [46].

Non-specific and antigen-specific immunological effects have been obtained in a considerable number of DC-treated AML patients. These effects include delayed-type hypersensitivity (DTH) skin test reactions, which essentially confirms the ability of the DCs to elicit T-cell-mediated immunity in vivo [52]. Other (non-specific) signs of the immunogenicity of DC therapy that have been observed include: increases in CD4^+^ and/or CD8^+^ T-cell frequencies during or after DC administration [49,53], enhanced activation of CD4^+^ T cells, as evidenced by their increased IFN-γ production following DC therapy [48], and elevations in plasma levels of immunostimulatory or T_H_1-polarizing cytokines (such as interleukin (IL)-2) [48,49,52]. Regarding the increased IFN-γ expression, it should be mentioned that IFN-γ can in turn have an effect on DCs. It is known that IFN-γ can induce IDO expression in subsets of DCs [54] and that this can induce tolerance in the DCs under specific circumstances. This might serve as an explanation why IDO-1 expression correlates with poor clinical outcome in patients with AML [55,56,57]. To date, studies have focused on the effects of IDO-expression in AML-blasts. Therefore, during the generation of AML-DCs derived from AML-blasts, their IDO-1 expression status might need to be taken into consideration. In contrast, the effect of IDO-expression on the phenotype and function of moDCs has not been studied yet. Several studies listed in Table 1, Table 2 and Table 3 have provided proof-of-principle that antigen-loaded DCs can induce leukemia antigen-specific T-cell immunity in patients with AML. Specific T-cell responses have been demonstrated by direct ex vivo tetramer analysis and/or following in vitro antigenic restimulation experiments towards the following AML-related tumor antigens: WT1 [33,35,36,39,44,51,52,53,58,59], PRAME [44,48,53,59], hTERT [39,60,61], and MUC1 [59]. It was also shown that DC-induced T-cell immune responses within a single patient can be directed against multiple antigens (e.g., WT1 and PRAME [44,53], or WT1 and hTERT [39]) and/or multiple epitopes a particular antigen (e.g., WT1_37–45_, WT1_126–134_, WT1_187–195_, and WT1_235–243_) [52]. This ability to induce multi-antigen- or multi-epitope-specific T-cell immunity is important, as this reduces the likelihood of tumor escape from T-cell recognition due to antigen loss (i.e., loss of expression of a single antigen/epitope) [62] or antigenic drift (i.e., mutations leading to epitope changes resulting in failure of the CTLs to recognize the original epitope) [63]. Theoretically, the use of DCs loaded with multiple antigens or AML lysates could also involve a higher risk of autoimmunity, for example, towards non-malignant cells that also express low levels of leukemia-associated antigens [64]. In clinical trials, treatment with multiple antigen-loaded DCs are well tolerated, and no or minor autoimmune reactions are normally observed [38]. Several studies have shown that DC therapy can also elicit leukemia antigen-specific T cells in the bone marrow compartment [39,59], which is of special importance in view of the observation that the bone marrow is the primary site where high-avidity AML-reactive CTLs reside [65].

In AML, DC vaccines have been applied in three different clinical settings: (a) in the context of HSCT, usually for treatment of relapsed AML after allogeneic HSCT (Table 1); (b) in an advanced disease setting, for example, for patients with refractory disease or relapsed AML for whom conventional treatment options have been exhausted (Table 2); and (c) after chemotherapy-induced remission of AML to prevent or delay relapse (Table 3). In the post-transplant relapse setting (Setting a), one study merits further discussion. Here, multi-genetically modified moDCs (Ad-siSSF DCs) were manufactured based on an adenovirus delivering: (i) secretory flagellin, a Toll-like receptor (TLR)-5 agonist inducing DC maturation; (ii) a survivin-MUC1 fusion protein, two leukemia-associated antigens; and (iii) SOCS1 shRNA, an RNA interference moiety overriding the intracellular immune checkpoint molecule SOCS1 [46]. Forty-eight patients with a post-transplant acute leukemia relapse (all AML, except for seven patients with acute lymphoblastic leukemia) were treated with either Ad-siSSF DCs or donor lymphocyte infusions (DLI). The vaccine was not only found to be safe but also induced a three-year OS of 48.9% compared with 27.5% in the DLI group. Thirteen out of 23 (57%) patients treated with the Ad-siSSF DCs achieved CR versus 12 out of 25 (48%) treated with DLI. In a second phase, 12 AML patients with early molecular relapse after HSCT were treated with Ad-siSSF DCs. Here, DC vaccination induced a CR rate of 83% (10 out of 12 patients). It should, however, be pointed out that the patients also received two subsequent infusions of cytokine-induced killer cells (CIKs), potentially contributing to the clinical effects of the DC vaccine. Moreover, all 12 patients were in early (molecular) relapse and efficacy would likely be lower in full-blown relapse [66,67]. This is supported by a mouse model of DC-based immunotherapy of AML [68], indicating that the therapeutic utility of DC vaccines is limited in the case of a high leukemic cell load.

In patients with relapsed/refractory AML (Setting b), clinical responses were usually limited to temporary disease stabilizations before further progression [39,41,48] and/or transient reductions in leukemic cell load [35,36,48]. The latter was evidenced either morphologically by demonstration of decreases in blast counts [48], or molecularly by demonstration of decreases in WT1 tumor marker transcript levels (as measured by quantitative reverse-transcriptase polymerase chain reaction (qRT-PCR) [35,36]. WT1 is a transcription factor used as a molecular marker for the monitoring of minimal residual disease in leukemia, especially in myeloid leukemias and myelodysplastic syndrome [69]. It is also a predictive factor of imminent relapse in AML patients, including those that received allo-SCT, even when other markers are not available [70,71,72,73]. Only one study [49] reported CR and partial remissions (PR) in the relapsed/refractory setting. It is important to note, however, that these patients also received chemotherapy and CIKs, making it difficult to draw conclusions about the effectiveness of DCs as a stand-alone treatment for advanced AML. In patients with advanced AML, immunosuppressive cells (T_reg_ cells and myeloid-derived suppressor cells (MDSCs)) may prevail over anti-tumor immune effector cells (CTLs and NK cells), explaining the higher likelihood of treatment failure when applying immunotherapy in the context of a high tumor burden [67,74].

The most obvious proof of clinical activity of DC vaccination in monotherapy has indeed been gathered in patients with low disease burden or MRD (Setting c). The concept is to administer DC vaccines as consolidation therapy to prevent or postpone relapse. Sustained and longer than usual CRs were reported in all post-remission DC vaccine studies, but the single-arm design of these studies precludes drawing firm conclusions on the true efficacy with respect to relapse prevention [28,40,43,44,50,51]. Nevertheless, several clinical trials have reported exceptionally long progression-free survival (PFS) times [42,44,45], indicating that DC vaccination can be an effective strategy to prevent/delay relapse. In the study by Rosenblatt et al. [42], 17 AML patients who achieved remission after chemotherapy, were vaccinated with moDCs fused to AML cells. This resulted in a 71% relapse-free survival at a median follow-up of 57 months. Moreover, the treatment was well tolerated and adverse events were transient and minor (grade 1–2 intensity). It should, however, be noted that the selection bias for long-term survivors requires careful interpretation of the data [42]. The achieved prevention of relapse is nonetheless remarkable when comparing to the treatment of patients aged 60–70 years with reduced intensity conditioning HSCT or chemotherapy alone resulting in three-year relapse-free survival of 68% and 19%, respectively [75]. In a study by Khoury et al. [45], 22 intermediate- or high-risk AML patients (median age of 58 years) were treated with human telomerase reverse transcriptase (hTERT)-expressing DCs. Given the central role of telomerase activity in maintaining self-renewal of leukemic stem cells, hTERT-DC vaccination may be ideally suited to target the small reservoir of residual leukemic stem cells that persist after chemotherapy. hTERT-DC vaccines administered in the post-remission setting were well tolerated and, after 52 months, 58% of the patients were free of disease recurrence. This compares favorably to the reported three-year relapse rates of 60% and 90% for patients with intermediate- and high-risk AML, respectively [76].

The group of Dr Felix Lichtenegger and Prof Marion Subklewe from Munich, Germany, used TLR-7/8-matured DCs loaded with *WT1*, *PRAME* and *CMVpp65* mRNA in 10 AML patients who were in remission after intensive chemotherapy, but at high risk of relapse. The vaccination proved to be safe and resulted in local inflammatory responses with dense T-cell infiltration. Increased antigen-specific CD8^+^ T cells were seen in peripheral blood for all three antigens. PFS was 1084 days, comparing favorably to a closely matched cohort from a patient registry of the same study group (Table 3). Median overall survival was not reached at the end of observation. In particular, excellent survival was seen in the immune responders (Ref. [44] and personal communication).

Our group has also shown that DC vaccination can confer an OS benefit in remission patients with AML. In a recently completed phase II clinical trial [8], we treated 30 AML patients with autologous, *WT1* mRNA-electroporated moDCs following standard induction chemotherapy; 27 of them were in CR and three were in PR. Two out of these three patients in PR were brought into CR by DC therapy. Most patients did not have morphologically demonstrable disease prior to the start of DC therapy but had evidence of residual disease at the molecular level (i.e., elevated *WT1* transcript levels in blood and/or marrow, as determined by qRT-PCR). In nine patients who had an increased level of the WT1 tumor marker at the start of DC therapy, *WT1* transcript levels returned to normal during DC vaccination, compatible with the induction of complete molecular remission (CMR). Five of these nine patients are still in CMR now more than five years after diagnosis and can be probably considered as cured. Apart from induction of morphological and/or molecular remission, four patients experienced disease stabilization for a period of time, a situation that is highly uncommon in AML given the aggressive behavior of this disease. The objective clinical response rate was 43%. PFS was significantly different in responders vs. non-responders. OS compared favorable to controls from the SEER and Swedish Acute Leukemia Registry, in patients ≤65 as well as >65 years, and was linked to the induction of WT1-specific CD8^+^ T-cell immunity [8]. Eleven out of 30 patients were alive in CR with a median OS from diagnosis of eight years (range 72.6–125.5 months), at the time of publication. These encouraging results have led us to embark on a follow-up randomized clinical trial comparing *WT1* mRNA-electroporated DC vaccination with standard-of-care in the post-remission setting of AML. The study is open for inclusion (Clinicaltrials.gov identifier NCT01686334).

## 3. Conclusions and Future Perspectives

Taken together, it can be concluded that DC-based immunotherapy has the potential to bring about demonstrable clinical responses in patients with AML. This holds particularly true in the post-remission setting of AML where treatment with DCs can produce durable remissions and prevent or delay relapse in some high-risk patients. Unfortunately, not all patients experience overt clinical benefit from this form of immunotherapy, underscoring the need to delve further into the possible reasons for therapeutic success or failure. In all studies listed in Table 1, Table 2 and Table 3 patients who failed to mount an immune response to DC vaccination had an inferior clinical outcome as compared to immune responders, indicating that the elicitation of (anti-leukemia) immunity by DCs is required to obtain a clinical response. For example, in our phase II clinical trial of WT1-targeted DC vaccination as a post-remission treatment for AML, only patients in whom DC vaccination elicited a poly-epitope WT1-specific CD8^+^ T-cell immune response experienced sustained CR [52,58]. There was also evidence in our study for a correlation between DC-induced NK cell activation and clinical activity [52]. As becomes evident from the data summarized in Table 1, Table 2 and Table 3 there is also a considerable number of patients who do not mount a clinical response despite the presence of DC-induced immune changes. One possible explanation for this observation is that the DCs currently used for immunotherapy are too weakly immunogenic to evoke clinically beneficial immune responses and/or that they do not induce the “right” type of immunity, i.e.:

- The immunostimulatory activity of the DCs may be too weak to induce high-avidity, long-lived leukemia-specific CTLs capable of mediating cytotoxicity of AML cells [33,39].
- The immunostimulatory activity of the DCs may be too weak to activate NK cells or γδ T-cells and harness innate immunity against AML cells [77,78,79,80,81,82].
- The immunostimulatory activity of the DCs may be too weak to overcome the immunosuppressive action of T_reg_ cells and MDSCs [35,36,51,83].
- The DCs used for therapy may favor the induction of a T_H_2 response over a T_H_1 response [84,85], which is otherwise the type of immunity that would be preferred in the setting of cancer immunotherapy [48].
- The DCs used for therapy may favor immune tolerance and produce undesired immune effects such as induction of T_reg_ cells and MDSCs [35,36,86].

These observations explain the impetus behind the many research efforts that are currently being undertaken to optimize the immunostimulatory properties of DCs in order to increase the likelihood of inducing protective anti-leukemia immunity in AML patients and, consequently, also the likelihood of therapeutic success [87]. One of the promising next-generation DC product candidates are IL-15-differentiated DCs [88]. In contrast to conventional IL-4 moDC vaccines, IL-15 DCs proved to be superior antigen-presenting cells, capable of direct tumoricidal activity [89], and, via expression of IL-15, capable of harnessing both NK cells and γδ T cells in the anti-tumor immune response [82,90].

Combining DC therapy with immune checkpoint targeting strategies, currently evoking a renaissance in the cancer immunotherapy field [91,92], is another avenue to unlock the full therapeutic potential of DC vaccines for AML. Moving beyond the combination of DC vaccines with systemic monoclonal antibodies, interceding programmed death (PD)-1/PD-L signaling in the DCs themselves reinforces the DC-mediated T cell and NK cell activation and prevents T_reg_ cell stimulation [91,92].

Another potential approach to enhance efficacy of DC therapy is combination with AML-specific monoclonal antibodies. Leukemia-specific mAb targets include CD33, CD123 and CD56, as reviewed in [93,94]. Especially ADCC-eliciting antibodies are interesting in the context of combination with DC vaccination, as ADCC-mediated killing results in the release of tumor neoantigens that can be taken up and cross-presented by tumor-residing DCs [95]. However, as of now, there are no studies combining such mAbs with DC therapy in AML.

Finally, there is an increasing interest to combine DC vaccination with conventional therapies, given the potential synergism between both. In our phase II clinical trial of *WT1* mRNA-electroporated DC vaccination, we observed unexpectedly high second remission rates and OS times to subsequent salvage treatment (i.e., chemotherapy and/or allogeneic HSCT) in vaccinated patients that experienced the first relapse. This may indicate that DC vaccination can potentiate the response to subsequent treatment, an observation that has also been made in the solid tumor vaccine field [67,96]. Hypomethylating agents (HMAs), which are being increasingly applied in the frontline treatment of elderly AML patients, have also shown synergistic activity with DC vaccination; one of the mechanisms underlying this synergism involves reduction of PD-1 expression on T cells and inhibition of MDSCs [97]. A phase II randomized clinical trial (Clinicaltrials.gov identifier NCT01686334) is currently ongoing to evaluate the effectiveness of combined HMA treatment and *WT1* mRNA-targeted DC vaccination.

## Figures and Tables

**Table 1:** Overview of DC (dendric cell) vaccine studies for AML (acute myeloid leukemia) in the post-HSCT setting.

	DC Type (Auto/Allo)	Antigen (Loading)	Immunological Effects	Clinical Effects
*n* = 1 [37]	CD34^+^ DCs	Apo-AML cells	Positive DTH	↓ AML cell load
	(allogeneic)	(pulsing)	↑ T-cell reactivity to DCs	(morphological)
*n* = 1 [32]	moDCs	WT1_235_	Positive DTH	Absent
	(allogeneic)	(pulsing)	↔ WT1-specific T cells	
*n* = 1 [47]	MoDCs *	Unloaded	Allo-MLR response to DCs	Absent
	(allogeneic)			
*n* = 1 [34]	moDCs	WT1_37;126;187_	Absence of WT1 response	Absent
	(allogeneic)	(pulsing)		
*n* = 2 [38]	moDCs	AML cell lysate	Positive DTH	Absent
	(autologous)	(pulsing)	↑ T-cell reactivity to DCs	
*n* = 19/23 [46]	MoDCs **	survivin/MUC1	ND	Induction of CR (13)
	(autologous)	(adenovirus)		Favorable OS (48.9% at 3 years)
*n* = 12 [46]	MoDCs **	survivin/MUC1	ND	Induction of CR (10)
	(autologous)	(adenovirus)		

Abbreviations: HSCT, hematopoietic stem cell transplantation; *n*, number of DC-treated patients; DC type, type of DC used; auto, DCs from autologous origin; allo, DCs from allogeneic origin; CD34^+^ DCs, DCs derived from CD34^+^ hematopoietic progenitor cells; moDCs, monocyte-derived DCs; *, in combination with donor lymphocyte infusions (DLI); **, in combination with cytokine-induced killer cells; Antigen, antigenic material used to load DCs; loading, antigen-loading method used; Apo-AML cells, apoptotic AML cells; WT1_37;126;187;235_, designated epitope derived from Wilms’ tumor 1 (WT1) antigen; MUC1, mucin 1; DTH, delayed-type hypersensitivity test; ↑, increase; ↔, steady state; allo-MLR, allogeneic mixed lymphocyte reaction; ND, no data; ↓, decrease; CR, complete remission; (number), number of patients in whom the designated clinical effect was observed; OS, overall survival.

**Table 2:** Overview of DC vaccine studies for AML in an advanced disease setting.

	DC Type (Auto/Allo)	Antigen (Loading)	Immunological Effects	Clinical Effects
*n* = 1 [41]	moDCs	AML cells	ND	Disease stabilization
	(allogeneic)	(fusion hybrids)		
*n* = 4 [39]	moDCs‖	Apo-AML cells	↑ AML-reactive T cells (2/4)	Disease stabilization (2/4)
	(autologous)	(pulsing)	↑ WT1/hTERT-specific T cells (1/1)	
*n* = 5 [48]	AML-DCs	NA	↑ PRAME-specific T cells (1/3)	Disease stabilization (1)
	(autologous)		↑ IFN-γ by CD4^+^ T cells (2/3)	↓ AML cell load (2)
			T_H_1/T_H_2 cytokine profile	(morphological)
*n* = 8 † [35,36]	moDCs ‖	WT1 peptide	↑ WT1-specific T cells	Disease stabilization (3)
	(autologous)	(pulsing)	(in clinical responders)	↓ AML cell load (2)
			↓ T_reg_ cells and MDSCs	(molecular)
			(in clinical responders)	
*n* = 21 [49]	AML-DCs **	NA	↑ CD4^+^ and CD8^+^ T cells	Induction of CR (6)
	(autologous)		↑ T_H_1 cytokines	Induction of PR (9)

Abbreviations: *n*, number of DC-treated patients; †, including two patients with acute lymphoblastic leukemia (ALL); DC type, type of DC used; auto, DCs from autologous origin; allo, DCs from allogeneic origin; moDCs, monocyte-derived DCs; ‖, in combination with systemic administration of the Toll-like receptor agonist OK432; AML-DCs, AML cell-derived DCs; **, in combination with cytokine-induced killer cells and low-dose chemotherapy (for further details, see [49]); Antigen, antigenic material used to load DCs; loading, antigen-loading method used; Apo-AML cells, apoptotic AML cells; NA, not applicable; WT1, Wilms’ tumor 1 antigen; ND, no data; ↑, increase; hTERT, human telomerase reverse transcriptase; PRAME, preferentially expressed antigen in melanoma; IFN-, interferon; T_H_1/T_H_2, T helper type 1 or 2; ↓, decrease; T_reg_, regulatory T cells; MDSCs, myeloid-derived suppressor cells; CR, complete remission; PR, partial remission; (number), number of patients in whom the designated immunological or clinical effect was observed.

**Table 3:** Overview of DC vaccine studies for AML in a post-remission setting.

	DC Type (Auto/Allo)	Antigen (Loading)	Immunological Effects	Clinical Effects
*n* = 3 [33]	moDCs ◊	WT1_235_	Positive DTH (2/3)	Disease stabilization (1/3)
	(autologous)	(pulsing)	↑ WT1-specific T cells (2/2)	↓ AML cell load (1/3)
			No ↑ γδ T cells	(morphological)
*n* = 5 [43,50]	moDCs	WT1/PRAME	Positive DTH (4)	Continued CR (21, 25, 33 m) (3)
	(autologous)	(mRNA EP)	↑ Ag-specific T cells (2)	
*n* = 5 [51]	AML-DCs	NA	Minimal or absent DTH	Continued CR (13–16 m) (2)
	(autologous)		↑ AML-reactive T cells (4/4)	
			↑ WT1-specific T cells (1/1)	
			No ↑ T_reg_ cells	
*n* = 5 [40]	moDCs	Apo-AML cells	ND	Continued CR (+13 m) (1)
	(autologous)	(pulsing)		
*n* = 12 [28]	AML-DCs	NA	Positive DTH	Disease stabilization (1)
	(allogeneic)		↑ WT1/PRAME-specific T cells	Disease stabilization (1)
				Favorable OS in patients without circulating blasts
*n* = 10/13 [44]	moDCs (autologous)	WT1/PRAME/CMVpp65 (mRNA EP)	Local immune response (10) ↑ Ag-specific T cells WT1 (2/10) PRAME (4/10) CMV (9/10)	Favorable RFS (1084 days vs. 396 days in matched cohort) Prolonged RFS and OS in immune responders
*n* = 17 [42]	moDCs	AML cells	↑ AML-reactive T cells (6)	Favorable RFS (71% at 57 m)
	(autologous)	(fusion hybrids)	↑ AML Ag-specific T cells (2)	
			(i.e., MUC1, WT1 or PRAME)	
*n* = 21 [45]	moDCs	hTERT	Positive DTH	Favorable RFS (58% at 52 m)
	(autologous)	(mRNA EP)	↑ hTERT-specfic T cells (11/19)	
*n* = 30 [8,52]	moDCs	WT1	Positive DTH	Induction of CMR (9)
	(autologous)	(mRNA EP)	↑ WT1-specific T cells	Disease stabilization (4)
			(in clinical responders)	Favorable RFS in responders
			NK activation (4/10)	Favorable OS

Abbreviations: *n*, number of DC-treated patients; DC type, type of DC used; auto, DCs from autologous origin; allo, DCs from allogeneic origin; moDCs, monocyte-derived DCs; ◊, pulsed with zoledronic acid in an attempt to induce γδ T-cell anti-leukemia immunity; AML-DCs, AML cell-derived DCs; Antigen, antigenic material used to load DCs; loading, antigen-loading method used; WT1_235_, designated epitope derived from Wilms’ tumor 1 (WT1) antigen; PRAME, preferentially expressed antigen in melanoma; mRNA EP, messenger RNA electroporation; NA, not applicable; Apo-AML cells, apoptotic AML cells; CMVpp65, Cytomegalovirus pp65 peptide; hTERT, human telomerase reverse transcriptase; DTH, delayed-type hypersensitivity test; ↑, increase; Ag, antigen; T_reg_, regulatory T cells; ND, no data; MUC1, mucin 1; NK, natural killer cell; ↓, decrease; CR, complete remission; CMR, complete molecular remission; RFS, relapse-free survival; OS, overall survival; (number), number of patients in whom the designated immunological or clinical effect was observed; (number m), follow-up time in months.
